# Mortality Statistics

**Published:** 1874-11

**Authors:** Ben. C. Miller

**Affiliations:** Sanitary Superintendent


***Chicago Mortality Report for September, 1874.*** Reported by Dr. BEN. C. MILLER, Sanitary Superintendent.

MORTALITY IN MONTH OF SEPTEMBER, 1874.

| | |
|---|---|
| Abscess | 1 |
| " lumbar | 1 |
| " hepatic | 1 |
| Accident, by burns | 1 |
| " by explosion | 1 |
| " by fracture of skull | 1 |
| " by fall | 2 |
| " kicked by horse | 1 |
| " run over by wagon | 1 |
| " by railroad | 3 |
| " by street cars | 1 |
| " by shooting | 2 |
| " by suffocation | 1 |
| " by drowning | 7 |
| Apoplexy | 6 |
| Asthma | 1 |
| Atelectasis pulmonum | 1 |
| Brain, congestion of | 8 |
| " hyperæmia of | 1 |
| " hemorrhage of | 1 |
| " inflammation of | 8 |
| " softening of | 3 |
| Bronchitis | 6 |
| " capillary | 2 |
| Cancer | 1 |
| " of abdomen | 1 |
| " of encephaloid | 1 |
| " of liver | 1 |
| " of stomach | 2 |
| Carbuncle on neck | 1 |
| Childbirth | 1 |
| Cholera infantum | 210 |

| | | | |
|---|---|---|---|
| Cholera morbus | 1 | Hydrocephalus | 9 |
| Colitis | 1 | Inanition | 22 |
| Consumption | 53 | Icterus | 2 |
| Convulsions | 85 | Kidneys, Bright's disease | 2 |
| Croup | 3 | " fatty degeneration of | 7 |
| " membranous | 2 | " inflammation of | 2 |
| Cyanosis | 1 | Laryngitis | 1 |
| Cystitis | 1 | Liver, disease of | 1 |
| Debility, general | 11 | " cirrhosis of | 1 |
| Delirium tremens | 2 | Lungs, congestion of | 3 |
| Diarrhœa | 39 | " gangrene of | 1 |
| " chronic | 6 | " hemorrhage of | 2 |
| Diphtheria | 4 | " emphysema of | 1 |
| Dropsy, general | 5 | " paralysis of | 1 |
| " abdominal | 1 | Manslaughter | 2 |
| Dysentery | 16 | Measles | 1 |
| Elephantiasis of leg | 1 | Meningitis | 12 |
| Enteritis | 26 | " cerebro-spinal | 6 |
| Entero-colitis | 15 | " tubercular | 4 |
| Erysipelas | 1 | Old age | 8 |
| Exhaustion | 2 | Paralysis | 6 |
| Fever, intermittent | 1 | Pericarditis | 1 |
| " scarlet | 16 | Peritonitis | 5 |
| " typhoid | 33 | " puerperal | 3 |
| Glands of throat, disease of | 1 | Pneumonia | 11 |
| " enlargement of | 1 | " typhoid | 1 |
| Gastritis | 3 | Pyæmia | 1 |
| Gastro enteritis | 7 | Rheumatism | 1 |
| Hemorrhage from abscess | 1 | " inflammatory | 1 |
| " internal | 1 | Scrofula | 2 |
| Hemorrhagica purpura | 1 | Small-Pox | 1 |
| Heart disease | 5 | Stomach, ulceration of | 2 |
| " hypertrophy of | 1 | Stomatitis | 1 |
| " organic disease of | 1 | Stricture of urethra | 1 |
| " ossification of | 1 | Suicide by laudanum | 1 |
| " rheumatism of | 1 | " by poison | 1 |
| " neuralgia of | 1 | " by shooting | 2 |
| " valvular disease of | 5 | Syphilis | 1 |
| Hepatitis | 2 | Tabes mesenterica | 21 |
| Hip, disease of | 1 | Teething | 8 |
| Hip-joint, disease of | 1 | Tetanus | 1 |
| Hernia | 1 | Whooping cough | 14 |
| Hydropericarditis | 1 | Total | 809 |
| Premature births, 14; Still births, 56. | | Total | 70 |

COMPARISON.

| | |
|---|---|
| Deaths in month of September, 1874 | 809 |
| " " August, 1874 | 1,220 |
| Decrease | 411 |
| Deaths in month of September, 1873 | 1,008 |
| Decrease | 199 |

AGES.

| | | | |
|---|---|---|---|
| Under one year | 327 | Four years to five | 11 |
| One year to two | 84 | Five " " ten | 29 |
| Two years to three | 26 | Ten " " twenty | 18 |
| Three " " four | 14 | Twenty " " thirty | 46 |

| | | | |
|---|---|---|---|
| Thirty years to forty | 50 | Seventy years to eighty | 13 |
| Forty " " fifty | 39 | Eighty " " ninety | 6 |
| Fifty " " sixty | 27 | Ninety " " one hundred | -- |
| Sixty " " seventy | 19 | Total | 809 |

| | | | |
|---|---|---|---|
| Colored | 10 | Males | 445 |
| White | 799 | Females | 364 |
| Total | 809 | Total | 809 |

Married, 159; Single, 650. Total 809

NATIVITIES, SEPTEMBER, 1874.

| | | | |
|---|---|---|---|
| Belgium | 1 | Ireland | 49 |
| Bohemia | 5 | Italy | 1 |
| Canada | 5 | Norway | 7 |
| Native—Chicago | 119 | Poland | 3 |
| Foreign, " | 422 | Scotland | 2 |
| United States, other parts | 98 | Sweden | 2 |
| Denmark | 3 | Switzerland | 1 |
| England | 17 | Wales | 1 |
| Germany | 63 | Unknown | 9 |
| Holland | 1 | Total | 809 |

Deaths daily, 27. Mean temperature, 66.8°. Rain fall, 3.93 inches.

MORTALITY BY WARDS, SEPTEMBER, 1874.

| Wards. | No. Deaths. | Pop. in 1872. | Percentage. |
|---|---|---|---|
| 1 | 4 | 398 | one death in ---- |
| 2 | 4 | 2,174 | " " ---- |
| 3 | 27 | 19,157 | " " 709 |
| 4 | 23 | 16,832 | " " 732 |
| 5 | 16 | 18,564 | " " 1,160 |
| 6 | 91 | 31,371 | " " 345 |
| 7 | 70 | 27,644 | " " 395 |
| 8 | 72 | 30,253 | " " 431 |
| 9 | 46 | 30,032 | " " 667 |
| 10 | 11 | 16,624 | " " 1,511 |
| 11 | 18 | 18,340 | " " 1,019 |
| 12 | 21 | 20,876 | " " 994 |
| 13 | 28 | 14,636 | " " 523 |
| 14 | 18 | 15,892 | " " 883 |
| 15 | 129 | 40,047 | " " 311 |
| 16 | 59 | 19,099 | " " 323 |
| 17 | 42 | 17,513 | " " 417 |
| 18 | 41 | 19,977 | " " 487 |
| 19 | 7 | 2,944 | " " ---- |
| 20 | 6 | 5,023 | " " ---- |
| | 733 | | |

Ratio of deaths to population in 1872, one death in 454⅛.

| | | | |
|---|---|---|---|
| No. deaths in Wards | 733 | St. Luke's Hospital | 1 |
| Accidents | 21 | Manslaughter | 2 |
| Foundlings' Home | 22 | Railroad Depot | 1 |
| Home for Friendless | 1 | Old Ladies' Home | 1 |
| Hospital Alexian Brothers | 3 | Suicides | 4 |
| Mercy Hospital | 6 | | |
| County Hospital | 13 | Total | 809 |
| Hospital for Women and Child'n | 2 | | |

